# Adherence to oxidative balance score is inversely associated with the prevalence of stroke: results from National Health and Nutrition Examination Survey 1999–2018

**DOI:** 10.3389/fneur.2024.1348011

**Published:** 2024-04-04

**Authors:** Jiarui Chen, Jianjian Liu, Zhaowen Gu, Jiayong Fan, Shuxin Lei, Qia Zhang, Kai Pan, Yongjie Wang

**Affiliations:** ^1^The Second Affiliated Hospital of Zhejiang University School of Medicine, Hangzhou, China; ^2^Clinical Research Center for Neurological Diseases of Zhejiang Province, Hangzhou, China; ^3^Key Laboratory of Precise Treatment and Clinical Translational Research of Neurological Diseases, Hangzhou, China

**Keywords:** stroke, NHANES, oxidative stress, oxidative balance score, prevention

## Abstract

**Introduction:**

The relationship between oxidative balance score (OBS), an emerging integrative metric for assessing individual redox homeostasis, and the prevalence of stroke in the general population remains unknown. We aimed to explore these relationships in the National Health and Nutrition Examination Survey (NHANES). We investigated the relationship between the oxidative balance score (OBS) and stroke prevalence using NHANES data from 1999–2018.

**Methods:**

We included eligible individuals from NHANES 1999–2018. OBS calculations were based on previously validated methods, and stroke diagnoses were based on self-reports in questionnaires. Multivariable logistic regression analyses were used to examine the independent associations of overall, dietary, and lifestyle OBS with stroke prevalence. In addition, restricted cubic spline (RCS), stratified analysis, and sensitivity analysis were used.

**Results:**

We included 25,258 participants aged 20–85 years, in which the prevalence of stroke was 2.66%. After adjusting for all confounders, overall and dietary OBS, but not lifestyle OBS, were inversely associated with the prevalence of stroke [odds ratios and 95% confidence intervals of 0.97 (0.96, 0.99) and 0.98 (0.96, 0.99) for overall and dietary OBS, respectively, both *p* < 0.05]. In addition, there was a dose-response relationship between overall and dietary OBS and stroke prevalence. The RCS showed that these relationships were linear. Stratified analyses indicated that socioeconomic status (SES) significantly influenced the relationship between all OBS and stroke prevalence.

**Conclusion:**

Dietary OBS, but not lifestyle OBS, had an inverse relationship with the prevalence of stroke in the general population. SES significantly influenced the protective effect of OBS against stroke. These findings emphasize the importance of integrated antioxidant properties from diet for stroke prevention.

## 1 Introduction

Stroke, including ischemic and hemorrhagic stroke, is the leading cause of disability and death worldwide (1). Global stroke statistics reveal that stroke is the second most common cause of disability and mortality, and its disease burden is substantial in both low- and high-income countries (2). Stroke is the disease leading to the highest disability-adjusted life-years lost in China, with approximately 2 million new cases each year (3). The incidence of stroke is highest in the elderly population; however, recent epidemiologic studies have shown that the incidence of stroke in young adults (<50 years of age) is increasing dramatically (4). Evidence-based findings suggest that up to 85% of strokes are preventable; therefore, identifying modifiable risk factors for stroke is a major instrument for stroke prevention and management of stroke populations (5).

Oxidative stress is an important component of the pathogenesis of stroke, which is inextricably associated with neuroinflammation and ischemia-reperfusion injury (6, 7). Dietary and other lifestyle modifications, which are recognized as central in stroke prevention, are closely linked to homeostasis of the individual’s redox state. There is a large body of evidence linking nutritional deficiencies and poor lifestyles such as obesity status and harmful alcohol consumption to stroke risk and prognosis (8–10). However, whether there are independent and/or combined effects of oxidative stress-related dietary components and lifestyle on the prevalence of stroke in the general population remains unstudied.

The oxidative balance score (OBS) is an emerging indicator in recent years for assessing the homeostasis of an individual’s redox balance, which consists of a dietary OBS, and a lifestyle OBS (11). OBS has demonstrated its clinical relevance in extensive epidemiologic studies and is associated with a variety of diseases such as cancer (12), chronic kidney disease (13), and depression (14). Exploring whether OBS is associated with the prevalence of stroke in the general population could help potential stroke prevention strategies based on OBS. We here explore these relationships using a nationwide population-based survey, the National Health and Nutrition Examination Surveys (NHANES).

## 2 Methods

### 2.1 Study population

NHANES was originally designed and conducted to collect information about the health and nutrition of the US household population, which is a major program of the National Center for Health Statistics (NCHS). NHANES is designed as a serial (2 years cycle from 1999 onwards), multistage, complex, probability-sampling cross-sectional study. The NHANES was reviewed and approved by the NCHS Ethics Review Board (ERB) (The ERB approval protocol numbers for each cycle can be found here: https://www.cdc.gov/nchs/nhanes/irba98.htm) and therefore do not require additional ethical review consent. In addition, written consent was obtained from all participants for this survey. More information is available on the official NHANES website.

**Figure 1:**
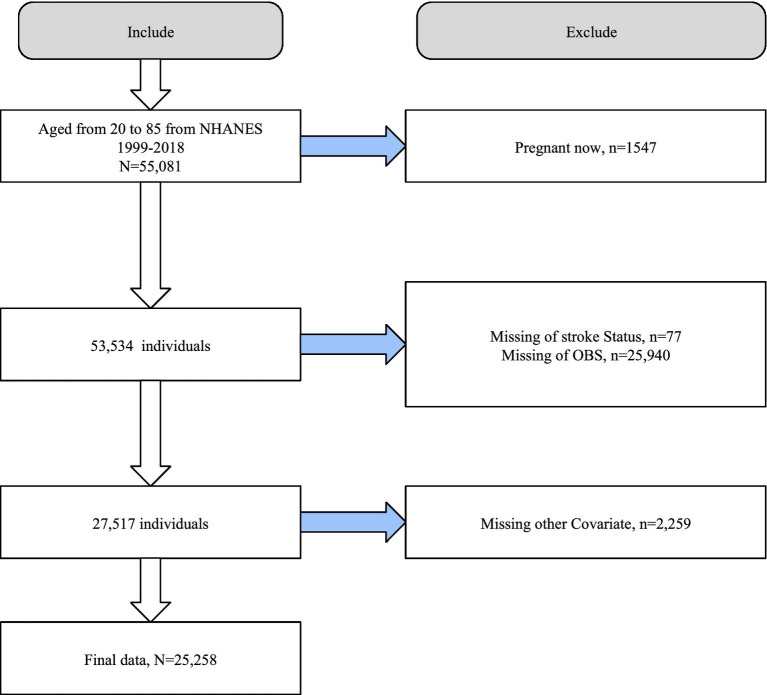
Flowchart of the study population selection process. OBS, oxidation balance score; NHANES, the National Health and Nutrition Examination Survey.

We first excluded pregnant participants (*n* = 1,547) among those aged 20–85 years (*n* = 55,081) in NHANES 1999–2018 (including 10 consecutive cycles). Next, we excluded participants with missing information on OBS (*n* = 25,940) and stroke (*n* = 77). We also ruled out those with missing covariates (*n* = 1,547). Finally, we included 25,258 participants for further analysis (Figure 1).

### 2.2 Definition of OBS

Numerous studies have explored the potential value of OBS using NHANES, and we used previously well-validated OBS components and calculations to define our OBS (15). The OBS consists of a dietary OBS composed of 16 dietary components and a lifestyle OBS composed of 4 lifestyle components. Of the 20 components, 5 of them were pro-oxidants (dietary iron and total fat intake, serum nicotine, BMI, alcohol consumption) and the others were antioxidants. In NHANES, two 24 h dietary review interviews were included (the first dietary recall interview was conducted in person and the second interview was conducted by telephone 3–10 days later), and we used the average intake from the two interviews. We did not include intake of nutrients from dietary supplements and medications based on the previous study. Physical activity was measured in metabolic equivalents [MET] based on the information in the questionnaire. We used serum nicotine as a measure of active and passive smoking to accurately reflect individual smoking levels.

We assigned scores to all OBS components based on a gender-specific approach. Antioxidant fractions were assigned scores of 0, 1, and 2 based on a lowest-to-highest distribution in tertiles, as opposed to pro-oxidants. We manually calculated the range of values corresponding to the tertiles of each component in the final included population. We assigned scores of 0, 1, and 2 to the >3, 2–3, and ≤2 drinks/day distributions of alcohol consumption in the past year for men, and scores of 0, 1, and 2 to the >2, 1–2, and ≤1 drinks/day for women. The component and score assignments of OBS are summarized in Table 1.

**Table 1:** Component and sex-specific scores assigned to OBS.

	Property	Male			Female		
		0	1	2	0	1	2
**Dietary OBS components**							
Dietary fiber (g/day)	A	<12.56	12.56–19.67	>19.67	<10.10	10.10–16.30	>16.30
Carotene (RE/day)	A	<98.62	98.62–305.85	>305.85	<98.06	98.06–383.92	>383.92
Riboflavin (mg/day)	A	<1.79	1.79–2.69	>2.69	<1.34	1.34–2.02	>2.02
Niacin (mg/day)	A	<20.64	20.64–29.75	>29.75	<14.51	14.51–21.85	>21.85
Vitamin B6 (mg/day)	A	<1.59	1.59–2.40	>2.40	<1.13	1.13–1.77	>1.77
Total folate (mcg/day)	A	<316.00	316.00–491.94	>491.94	<251.50	251.0–388.5	>388.5
Vitamin B12 (mcg/day)	A	<3.36	3.36–6.20	>6.20	<2.22	2.22–4.21	>4.21
Vitamin C (mg/day)	A	<42.40	42.40–113.20	>113.20	<38.01	38.01–98.40	>98.40
Vitamin E (ATE) (mg/day)	A	<5.82	5.82–9.41	>9.41	<4.53	4.53–7.51	>7.51
Calcium (mg/day)	A	<645.50	645.50–1071.50	>1071.50	<499.23	499.23–848.78	>848.78
Magnesium (mg/day)	A	<257.00	257.00–361.05	>361.05	<187.00	187.00–283.21	>283.21
Zinc (mg/day)	A	<9.75	9.75–15.10	>15.10	<6.73	6.73–10.74	>10.74
Copper (mg/day)	A	<1.12	1.12–1.57	>1.57	<0.85	0.85–1.28	>1.28
Selenium (mcg/day)	A	<94.94	94.94–141.75	>141.75	<67.75	67.75–99.45	>99.45
Total fat (g/day)	P	>107.42	69.83–107.42	<69.83	>75.78	50.98–75.78	<50.98
Iron (mg/day)	P	>19.16	12.88–19.16	<12.88	>14.32	9.65–14.32	<9.65
**Lifestyle OBS components**							
Physical activity (MET-minute/week)	A	<417.90	417.90–1135.40	>1135.40	<270.67	270.67–843.27	>843.27
Alcohol (drinks/day)	P	>3 drinks/day	2–3 drinks/day	≤2 drinks/day	≤2 drinks/day	1–2 drinks/day	≤1 drinks/day
Body mass index (kg/m^2^)	P	>29.17	25.55–29.17	<25.55	>28.63	23.75–28.63	<23.75
Cotinine (ng/mL)	P	>1.13	0.04–1.13	<0.04	>0.17	0.04–0.17	<0.04

OBS, oxidation balance score; A, antioxidant; P, pro-oxidant.

### 2.3 Definition of stroke

We defined an individual’s stroke status based on the question in the NHANES questionnaire: “Has a doctor or other health professional ever told you that you had a stroke?” (16). The diagnosis of such questionnaire-based self-reported illnesses has been shown to have good agreement and has been widely used in NHANES-related epidemiologic studies (17).

### 2.4 Covariate

We selected several important potential covariates based on previous studies, including age, gender (male or female), ethnicity, education level, marital status (single or non-single), family income to poverty (PIR), total daily energy intake (kcal/day), diabetes, and hypertension. The ethnicity consists of Mexican American, non-Hispanic black, non-Hispanic white, other Hispanic, or other races. The educational level of an individual was categorized as <high school, high school, and >high school. A diagnosis of diabetes was based on one of a doctor saying someone has diabetes, a blood glucose/glucose tolerance test that meets the American Diabetes Association’s criteria (18), or being on anti-diabetic medication. Hypertension was diagnosed by self-reported hypertension, a blood pressure ≥130/85 mmHg, or taking antihypertensive medication.

### 2.5 Statistical analysis

All analyses were performed using EmpowerStats (X&Y Solutions, Inc., Boston, MA) and R software 4.2.3. Given the complex design of the NHANES and in an effort to make our sample representative of the entire US population, we weighted our analyses accordingly to the NHANES analysis guidelines (19). In the baseline analysis, the continuous variables (mean ± standard error) and categorical variables (percentage) were used to characterize the study population. The student’s *t*-test for continuous variables or chi-square test for categorical variables was applied.

In the multivariable adjusted logistic regression analysis, we constructed three models. The crude model was not adjusted for any covariates. Adjusted model 1 was a partially adjusted model that adjusted for age, gender, race, marital status, PIR, education level, and total energy intake. Adjusted model 2 was further adjusted for diabetes and hypertension based on adjusted model 1.

To explore the potential nonlinear relationship between OBS and stroke, we performed smooth curve fitting using restricted cubic spline (RCS). Finally, we used stratified analyses to explore the consistency of the results within subgroups for each variable and performed sensitivity analyses using tertiles and quintiles of OBS to indicate whether the results were stable. In all analyses, *p* < 0.05 was considered statistically significant.

## 3 Results

### 3.1 Baseline characteristics

Of the 25,258 participants included (mean age 46.04 years, 51.29% male), stroke was diagnosed in 673 subjects, corresponding to a prevalence of 2.66%. We first performed a baseline analysis of the included population according to the quartiles of the OBS. We found that as quartiles of OBS rose, participants had significantly higher PIR, energy intake, proportion of non-Hispanic white population, proportion of non-singles, and education levels, while diabetes, hypertension, and stroke populations were significantly lower. However, participants’ age and sex proportions did not change significantly with OBS. In addition, dietary and lifestyle OBS increased significantly with increasing quartiles of OBS (Table 2).

**Table 2:** Baseline characteristics of the included population, grouped according to quartiles of the OBS.

Variable	Total	OBS Q1	OBS Q2	OBS Q3	OBS Q4	*p*-value
Age, year	46.04 ± 0.22	45.76 ± 0.28	46.57 ± 0.31	46.00 ± 0.35	45.84 ± 0.31	0.12
PIR	3.20 ± 0.03	2.78 ± 0.04	3.12 ± 0.03	3.30 ± 0.04	3.48 ± 0.04	<0.0001
OBS dietary	17.07 ± 0.09	7.57 ± 0.04	14.04 ± 0.03	19.18 ± 0.02	24.67 ± 0.03	<0.0001
OBS lifestyle	4.80 ± 0.02	4.27 ± 0.03	4.62 ± 0.03	5.07 ± 0.03	5.11 ± 0.03	<0.0001
OBS	21.87 ± 0.10	11.75 ± 0.05	18.64 ± 0.03	24.04 ± 0.02	30.01 ± 0.04	<0.0001
Energy intake, kcal/day	2182.34 ± 7.52	1522.68 ± 9.59	1957.53 ± 11.30	2261.48 ± 12.31	2775.79 ± 15.41	<0.0001
Sex						0.27
Male	13,285 (51.29)	3,450 (52.75)	3,362 (51.37)	2,979 (50.59)	3,494 (50.73)	
Female	11,973 (48.71)	2,740 (47.25)	3,044 (48.63)	2,829 (49.41)	3,360 (49.27)	
Race						<0.0001
Mexican American	3,898 (6.71)	957 (6.62)	991 (6.87)	895 (6.71)	1,055 (6.65)	
Non-Hispanic black	4,707 (8.96)	1,594 (13.85)	1,296 (9.98)	903 (7.28)	914 (5.96)	
Non-Hispanic white	12,745 (73.92)	2,811 (68.83)	3,126 (72.34)	3,047 (75.65)	3,761 (77.46)	
Other Hispanic	1,821 (4.67)	448 (5.42)	466 (4.87)	419 (4.29)	488 (4.28)	
Other race	2,087 (5.74)	380 (5.28)	527 (5.94)	544 (6.07)	636 (5.64)	
Marital status						<0.0001
Non-single	15,797 (66.21)	3,624 (61.42)	4,016 (66.16)	3,706 (67.61)	4,451 (68.54)	
Single	9,461 (33.79)	2,566 (38.58)	2,390 (33.84)	2,102 (32.39)	2,403 (31.46)	
Education						<0.0001
<High school	1,963 (3.54)	736 (5.71)	539 (3.95)	352 (2.73)	336 (2.32)	
High school	8,862 (31.89)	2,634 (41.37)	2,357 (34.60)	1,910 (29.72)	1,961 (24.61)	
>High school	14,433 (64.57)	2,820 (52.91)	3,510 (61.45)	3,546 (67.55)	4,557 (73.07)	
Diabetes						<0.0001
No	21,643 (89.53)	5,131 (88.27)	5,432 (88.44)	5,021 (89.95)	6,059 (91.00)	
Yes	3,615 (10.47)	1,059 (11.73)	974 (11.56)	787 (10.05)	795 (9.00)	
Hypertension						<0.0001
No	15,368 (65.82)	3,470 (63.19)	3,801 (63.79)	3,617 (66.70)	4,480 (68.67)	
Yes	9,890 (34.18)	2,720 (36.81)	2,605 (36.21)	2,191 (33.30)	2,374 (31.33)	
Stroke						0.001
No	24,585 (98.07)	5,958 (97.50)	6,229 (97.78)	5,676 (98.42)	6,722 (98.43)	
Yes	673 (1.93)	232 (2.50)	177 (2.22)	132 (1.58)	132 (1.57)	

OBS, oxidation balance score; PIR, family income to poverty.

We summarized the baseline characteristics of the population grouped according to stroke status in Supplementary Table S1. There were significant differences in age, PIR, total energy intake, sex, race, education, diabetes, and hypertension status in individuals with stroke compared to the stroke-free population, while there were no differences in marital status. Of note, OBS, dietary OBS, and lifestyle OBS were all lower in people with stroke (*p* < 0.0001, < 0.0001, and = 0.005, respectively).

### 3.2 Multivariable logistic regression analysis

To explore whether OBS (including dietary and lifestyle OBS) is independently associated with the odds of stroke in the general population, we developed three multivariable models with stepwise adjustment. We treated OBS as continuous and categorical variables in quartiles, respectively, where the number of participants and the range of values for all quartiles of OBS were listed in Supplementary Table S2. First, when not adjusting for any confounders, we found that dietary OBS but not lifestyle OBS was inversely associated with stroke. When used as a continuous variable, each unit increase in dietary OBS was associated with 3% lower odds of stroke [odds ratio (OR) and 95% confidence interval (CI) = 0.97 (0.96, 0.98), *p* < 0.0001]. Similarly, overall OBS showed a similar trend [OR and 95% CI = 0.97 (0.96, 0.98), *p* < 0.0001]. However, there was no significant association for lifestyle OBS [OR and 95% CI = 1.01 (0.95, 1.07) *p* = 0.7358]. When treated as categorical variables, both dietary and overall OBS showed a significant dose-response relationship with stroke risk (both *p* for trend <0.05). Compared to Q1, dietary OBS was associated with a 40% reduction in the prevalence of stroke in both Q3 and Q4, while similarly, overall OBS was associated with a 32 and 37% reduction in risk in Q3 and Q4, respectively. However, we did not observe similar changes in lifestyle OBS.

We then adjusted for age, sex, race, education level, marital status, PIR, and total daily energy intake in adjusted model 1. Similar associations with stroke prevalence were still observed in diet and overall OBS. Notably, we found that lifestyle OBS was also negatively associated with stroke prevalence [OR and 95% CI = 0.92 (0.86, 0.98), *p* = 0.0119] and exhibited a dose-response relationship (*p* for trend = 0.0063).

Finally, we additionally adjusted for diabetes and hypertension status based on model 1. In the fully adjusted model, dietary OBS remained independently and inversely associated with stroke prevalence, with each unit increase in OBS associated with a 2% reduction in stroke risk. Similarly, we observed a dose-response relationship between dietary OBS and stroke risk (*p* for trend = 0.0271), with dietary OBS at Q3 associated with a 31% reduction in stroke prevalence, and Q4 also marginally associated with a reduced risk of stroke compared to Q1 (OR = 0.73, *p* = 0.0677). A similar trend was observed for overall OBS. A 1-unit increase in an individual’s overall OBS was associated with a 3% reduction in stroke risk, and Q3 and Q4 OBS were associated with 28 and 32% reductions in stroke prevalence. However, we found that the association between lifestyle OBS and stroke risk disappeared after adjusting for all confounders (all *p* > 0.05) (Table 3).

**Table 3:** Multivariable-adjusted logistic regression models examining the relationship between OBS and stroke prevalence.

	Crude Model OR (95% CI) *p*-value	Adjusted model 1 OR (95% CI) *p*-value	Adjusted model 2 OR (95% CI) *p*-value
OBS dietary	0.97 (0.96, 0.98) <0.0001	0.98 (0.96, 1.00) 0.0164	0.98 (0.96, 0.99) 0.0128
**OBS dietary quartile**			
Q1	Ref.	Ref.	Ref.
Q2	0.86 (0.66, 1.13) 0.2733	0.93 (0.69, 1.24) 0.6090	0.92 (0.68, 1.23) 0.5641
Q3	0.60 (0.46, 0.80) 0.0005	0.70 (0.50, 0.96) 0.0297	0.69 (0.50, 0.96) 0.0273
Q4	0.60 (0.46, 0.78) 0.0002	0.74 (0.52, 1.04) 0.0817	0.73 (0.51, 1.02) 0.0677
*p* for trend	<0.0001	0.0321	0.0271
OBS lifestyle	1.01 (0.95, 1.07) 0.7358	0.92 (0.86, 0.98) 0.0119	0.96 (0.89, 1.02) 0.2011
**OBS lifestyle quartile**			
Q1	Ref.	Ref.	Ref.
Q2	1.29 (0.96, 1.73) 0.0921	1.01 (0.75, 1.37) 0.9427	1.05 (0.77, 1.43) 0.7582
Q3	1.27 (0.92, 1.75) 0.1506	0.85 (0.60, 1.20) 0.3609	0.93 (0.65, 1.32) 0.6830
Q4	1.08 (0.80, 1.47) 0.6039	0.70 (0.51, 0.96) 0.0267	0.81 (0.59, 1.12) 0.2004
*p* for trend	0.8309	0.0063	0.0977
OBS	0.97 (0.96, 0.98) <0.0001	0.97 (0.96, 0.99) 0.0017	0.97 (0.96, 0.99) 0.0019
**OBS quartile**			
Q1	Ref.	Ref.	Ref.
Q2	0.87 (0.67, 1.14) 0.3056	0.88 (0.66, 1.18) 0.4015	0.88 (0.66, 1.17) 0.3754
Q3	0.68 (0.51, 0.90) 0.0076	0.72 (0.53, 0.99) 0.0478	0.72 (0.53, 0.99) 0.0479
Q4	0.63 (0.48, 0.81) 0.0005	0.68 (0.49, 0.95) 0.0271	0.68 (0.49, 0.96) 0.0276
*p* for trend	0.0001	0.0157	0.0169

OBS, oxidation balance score. The crude model was not adjusted for any covariates. Adjusted model 1 was adjusted for age, gender, race, marital status, PIR, education level, and total energy intake. Adjusted model 2 was further adjusted for diabetes and hypertension based on adjusted model 1.

### 3.3 RCS modeling and smoothed curve fitting

**Figure 2:**
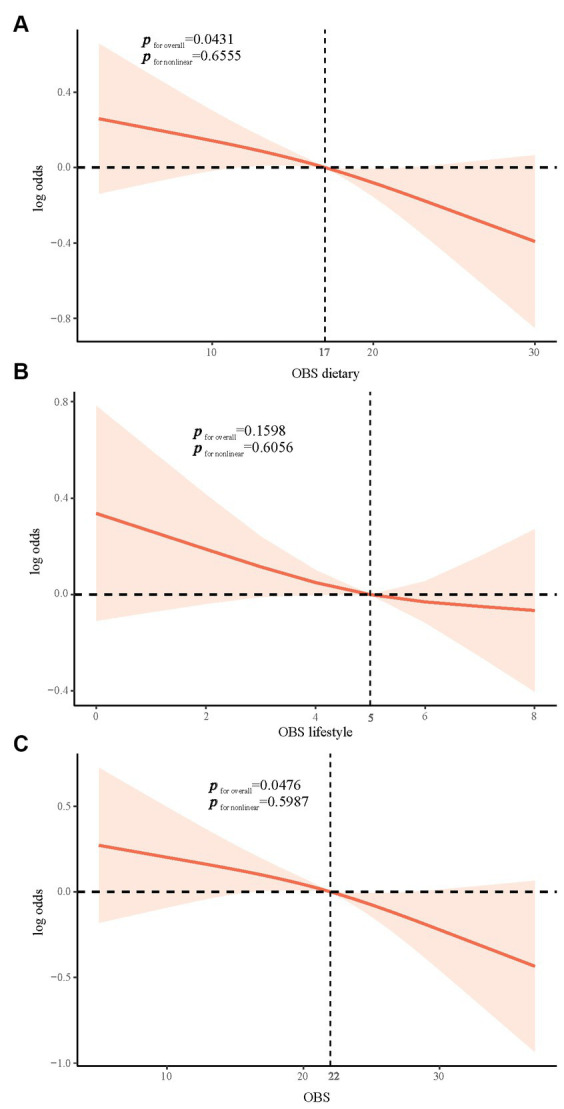
RCS model exploring nonlinear relationships. Linear associations in dietary and overall OBS are shown in **(A and C)**, No significant association in lifestyle OBS is shown in **(B)**. OBS, oxidation balance score; RCS, restricted cubic spline.

To explore whether there was a nonlinear relationship between all OBS and stroke risk, we performed an exploration using the RCS model. We found that both dietary and overall OBS were linearly associated with stroke risk in the general population (*p* nonlinear = 0.6555 and 0.5987, respectively). Furthermore, the development of stroke would likely be at a relatively low risk after dietary and overall OBS exceeded 17 and 22 points, respectively. Consistently, we found no significant association between lifestyle OBS and the prevalence of stroke (Figure 2).

### 3.4 Stratified analysis

**Figure 3:**
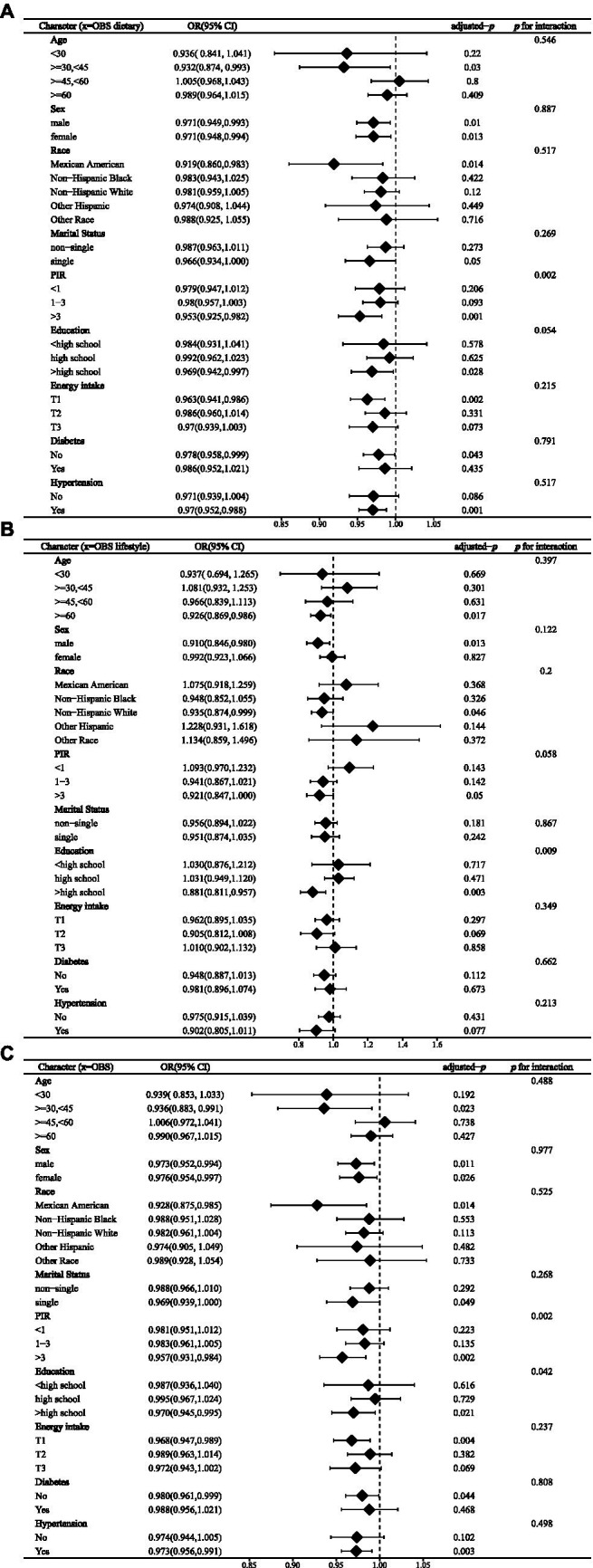
Stratified analysis of OBS components and stroke prevalence: interaction with PIR and education level in dietary OBS **(A)**, lifestyle OBS **(B)**, and overall OBS **(C)**. PIR, family income to poverty; OR, odds ratio.

In stratified analyses, we found no multiplicative interaction between most of the variables and OBS (*p* for interaction >0.05). However, we nonetheless found several factors that may influence the relationship between OBS and stroke. In the association between dietary OBS and the prevalence of stroke, we found a significant interaction between PIR and this relationship (*p* for interaction = 0.002), and a marginal effect of education level (*p* for interaction = 0.054) (Figure 3A). Similarly, we observed the effect of PIR and education level on the relationship in stratified analyses for lifestyle OBS (*p* for interaction = 0.058 and 0.009 for PIR and education level, respectively) and overall OBS (*p* for interaction = 0.002 and 0.042 for PIR and education level, respectively) (Figures 3B,C). The associations of OBS, dietary OBS, and lifestyle OBS with the prevalence of stroke were only present in participants with a PIR >3 and an education level above high school.

### 3.5 Sensitivity analysis

We conducted sensitivity analyses by treating all OBS as categorical variables in tertiles or quintiles to verify the stability of the findings. We obtained similar results, although the dose-response relationship was lost for dietary OBS under tertiles (*p* for trend = 0.1707). This may indicate that the association of dietary OBS on stroke was significant but relatively flat and that more significant changes are needed to be associated with a reduction in stroke prevalence. Similarly, lifestyle OBS remained unassociated with stroke prevalence (all *p* > 0.05) (Supplementary Tables S3, S4).

## 4 Discussion

### 4.1 Main findings

In this nationally representative population-based study, we found that an emerging measure of individual redox homeostasis, OBS, was inversely associated with the prevalence of self-reported stroke. Specifically, dietary OBS was significantly associated with stroke, but not lifestyle OBS. The RCS model revealed that OBS and dietary OBS were linearly associated with stroke prevalence in the general population. Another important finding was that participants’ socioeconomic status (SES) was a key factor influencing these relationships, including lifestyle OBS with stroke prevalence. Our study suggests that higher diet-based antioxidant profiles in the general population are associated with a reduction in the development of stroke, and these findings emphasize the plausibility of stroke prevention by adjusting daily dietary antioxidant levels.

### 4.2 Comparison with other studies

Diet/nutrition-related strategies have been shown to have a significant impact on stroke prevention in the general population. Of these, the Mediterranean diet has been the most extensively studied for stroke prevention. The Mediterranean diet is a healthy, light, yet nutritionally complete diet. It is characterized by an adequate intake of fruits, vegetables, and whole grains, and also include legumes/nuts, skim milk, olive oil and some fish, as well as small amounts of red meat, salt, and carbohydrates (20). The Mediterranean diet has proven benefits for cardiovascular health as well as disease prevention (21). Adherence to the Mediterranean diet has been reported to reduce the risk of first stroke (22) and post-stroke cardiometabolic health (23). In addition, extensive body of research suggests that a variety of indicators reflecting dietary habits or quality are also associated with the risk of developing stroke. A recent epidemiologic study using NHANES indicated that stroke survivors had lower levels of nutrient intake and overall diet quality (as indicated by the Healthy Eating Index-2015) than a matched control population (24). Another indicator of diet quality, the Dietary Inflammation Index, has also been shown to be associated with risk of stroke (25), post-stroke depression (26), and increased carotid plaque vulnerability (27). Similarly, a meta-analysis incorporating 12 prospective cohort studies demonstrated that Dietary Approaches to Stop Hypertension dietary patterns were inversely associated with the risk of stroke development (relative risk 0.88, 95% CI 0.83–0.93) (28). These studies underscore the importance of dietary/nutritional factors in stroke prevention.

We showed for the first time that OBS was independently associated with the prevalence of stroke in the general population in a large, real-world epidemiologic study. The OBS is an emerging scoring system composed of multiple dietary and lifestyle sources of antioxidants and pro-oxidants that reflect an individual’s exogenous redox homeostasis, with a higher level representing an individual’s overall antioxidant profile at a higher level (11). Although the composition of OBS components in the current literature is diverse, OBS has been shown in numerous observational studies to be associated with cardiovascular risk factors (29), multiple cancers (12, 30), and all-cause/factor-specific mortality (31). In our study, the components of OBS were determined based on previous studies using NHANES (15, 32), thus ensuring reliability and applicability. Our OBS consists of 16 dietary (including macro- and micronutrients) and 4 lifestyle antioxidants and pro-oxidants, all of which have been shown in the literature to have potential antioxidant/pro-oxidant properties (11, 32). The comprehensive inclusion of dietary and lifestyle sources of antioxidants and pro-oxidants allowed for a more adequate assessment of exogenous redox homeostasis in individuals by the OBS in our study.

While no studies have explored the relationship between OBS and stroke risk, numerous clinical studies have shown that dietary antioxidants are strongly associated with the development of stroke, albeit with controversy. Earlier studies have shown that the intake of certain dietary antioxidants is associated with a reduced risk of stroke (33, 34). However, recent evidence suggests that dietary antioxidants are not associated with stroke risk and even increase the risk of certain stroke types (35, 36). However, these studies only examined the relationship between one or specific dietary antioxidants and the risk of stroke, unlike the integrated assessment of dietary antioxidants and pro-oxidants in our study. Recent studies have similarly used NHANES to show inverse associations of otherwise measured dietary antioxidant properties with risk of stroke (37) and post-stroke depression/all-cause mortality (38), consistent with our study.

Surprisingly, we did not find an association between lifestyle OBS and stroke prevalence. Lifestyle OBS consists of BMI, physical activity, alcohol consumption, and serum nicotine. A large meta-analysis that included 4.43 million people showed a “J” shaped relationship between BMI and the risk of stroke, with BMI >25 kg/m^2^ being associated with an increased risk of stroke (39). Similarly, heavy alcohol consumption was associated with an increased risk of stroke, whereas light-moderate alcohol intake was associated with a decreased risk of stroke (10). Smoking or electronic nicotine delivery systems use have also been shown to be associated with the risk of stroke (40, 41), although the association between serum nicotine levels and the risk of stroke remains unclear. Physical activity, on the other hand, is recognized as an effective means in stroke prevention (42). We were unable to provide a specific explanation at this time, but this may be due to the heterogeneity of our inclusion population compared to previous studies, as well as the differences in lifestyle OBS as a composite measure compared to individual lifestyles. In addition, we can draw insights from the stratified analysis. Although lifestyle OBS was not associated with stroke prevalence in the overall population, we observed significant associations in specific subgroups such as those ≥60 years of age, men, non-Hispanic white population, and those with >high school education. This suggests that the impact of an individual’s lifestyle OBS on the prevalence of stroke in the general population is age-, sex-, race-, and education-specific. It also highlights the need for lifestyle-based stroke prevention strategies to take these demographic and SES factors into account.

Another important finding of our study was that SES significantly influenced the relationship between OBS (including dietary and lifestyle OBS) and stroke development. A recent study using NHANES showed a significantly inverse association of PIR with stroke risk (43). Our study showed that PIR significantly/marginally influenced the relationship between OBS and stroke risk, and that a protective effect of OBS was present only in participants with a PIR >3. Another study using NHANES showed that poverty was more common among stroke survivors and was associated with lower dietary quality, which may also partly explain the protective effect of OBS only among those with higher incomes (24). In addition, educational attainment has also been shown to be significantly associated with stroke risk in previous studies (44, 45). These findings were consistent with previous studies indicating a significant effect of SES on individuals with stroke, either alone or by influencing the relationship.

### 4.3 Potential mechanisms

OBS was revealed in previous studies to be associated with oxidative stress and inflammatory biomarkers in the blood. Multiple previous studies have shown that OBS was correlated with levels of biomarkers reflecting systemic oxidative stress levels such as F2-isoprostanes, fluorescent oxidative products, and γ-glutamyltransferase (46–49). OBS has also been revealed to have a possible negative correlation with several biomarkers of systemic inflammation such as C-reactive protein, and interleukin-6 levels (47, 50). Thus, OBS may serve as a validated measure of the redox status of an individual’s diet and lifestyle, which then indirectly reflects the level of systemic oxidative stress and chronic inflammation, which are critical in the pathogenesis of stroke (6, 51).

### 4.4 Strengths and limitations

Our study has several strengths. Firstly, this represents a pioneering real-world exploration of the relationship between an emerging composite of antioxidant and pro-oxidant metrics reflecting diet and lifestyle and the prevalence of stroke in the general population. These findings may illuminate potential pathways for the implementation of future OBS-based stroke prevention strategies, contributing to the primordial prevention of stroke. This has important public health implications for reducing the disease burden of stroke. Secondly, our study made extensive adjustments for numerous potential confounders, thereby minimizing the impact of confounding. Our study was based on a nationwide epidemiological survey, and the use of appropriate weighting methods allowed our sample to be representative of the overall population. This ensures the representativeness and generalizability of our findings.

However, there are weaknesses to be acknowledged. Firstly, due to the cross-sectional nature of the study, we were unable to establish causality and may still be subject to unadjusted residual confounders. Secondly, the diagnosis of stroke in our study was reliant on self-reporting via questionnaires, which could introduce information bias. Nonetheless, a large body of previous NHANES studies that have utilized questionnaires for diagnosis have reported convincing agreement. Additionally, our analysis did not include the intake of nutrients from dietary supplements and medications, consistent with previous research approaches. This decision was based on prior studies suggesting that the inclusion of such data does not significantly alter the associations under investigation. However, it is important to recognize that this could affect the estimation of total nutrient intake and potentially influence the observed associations. Future research could benefit from incorporating these factors to provide a more comprehensive assessment.

In conclusion, while our findings provide valuable insights, they should be interpreted with caution. Further research, especially large prospective cohort studies, is needed to substantiate and expand upon our conclusions.

## 5 Conclusion

In a nationally representative population-based study, we found that overall OBS and dietary OBS, but not lifestyle OBS, were inversely associated with the prevalence of stroke in the general population. SES, including PIR and educational attainment, significantly influenced these associations. These findings may contribute to further exploration of stroke prevention strategies targeting OBS.

## Data availability statement

Publicly available datasets were analyzed in this study. This data can be found here: Centers for Disease Control and Prevention (CDC), National Center for Health Statistics (NCHS), National Health and Nutrition Examination Survey (NHANES), https://wwwn.cdc.gov/nchs/nhanes/Default.aspx, NHANES 1999–2018.

## Ethics statement

Ethical review and approval was not required for the study on human participants in accordance with the local legislation and institutional requirements. Written informed consent from the patients/participants or patients/participants’ legal guardian/next of kin was not required to participate in this study in accordance with the national legislation and the institutional requirements.

## Author contributions

JC: Writing – original draft, Conceptualization, Methodology, Formal analysis, Software. JL: Methodology, Formal analysis, Writing – review & editing. ZG: Writing – original draft, Conceptualization, Methodology. JF: Methodology, Formal analysis, Writing – review & editing. SL: Writing – review & editing, Formal analysis. QZ: Writing – review & editing. KP: Writing – review & editing. YW: Writing – review & editing, Conceptualization, Methodology, Project administration, Funding acquisition.
