# Supplementary material for: Adherence to oxidative balance score is inversely associated with the prevalence of stroke: results from National Health and Nutrition Examination Survey 1999–2018
Source: Front Neurol. 2024 Apr 4;15:1348011. doi: 10.3389/fneur.2024.1348011 (PMC11024455; doi:10.3389/fneur.2024.1348011)
Supplement: Supplementary file 1 [file Table_1.DOCX]

Supplementary Material

# Supplementary Tables

**Table S1.** Baseline characteristics grouped according to stroke status.

| **Variable** | **Total** | **Non-Stroke** | **Stroke** | **P value** |
| --- | --- | --- | --- | --- |
| **Age, year** | 46.04±0.22 | 45.74±0.22 | 61.48±0.76 | < 0.0001 |
| **PIR** | 3.20±0.03 | 3.21±0.03 | 2.68±0.08 | < 0.0001 |
| **OBS.dietary** | 17.07±0.09 | 17.10±0.09 | 15.69±0.32 | < 0.0001 |
| **OBS.lifestyle** | 4.80±0.02 | 4.81±0.02 | 4.58±0.08 | 0.005 |
| **OBS** | 21.87±0.10 | 21.90±0.10 | 20.42±0.34 | < 0.0001 |
| **Energy intake, kcal/day** | 2182.34±7.52 | 2187.01± 7.48 | 1945.02±44.24 | < 0.0001 |
| **Sex** |  |  |  | 0.01 |
| male | 13285(51.29) | 12934(51.41) | 351(45.10) |  |
| female | 11973(48.71) | 11651(48.59) | 322(54.90) |  |
| Race |  |  |  | 0.001 |
| Mexican American | 3898(6.71) | 3823(6.77) | 75(3.94) |  |
| Non-Hispanic Black | 4707(8.96) | 4552(8.89) | 155(12.09) |  |
| Non-Hispanic White | 12745(73.92) | 12370(73.88) | 375(75.70) |  |
| Other Hispanic | 1821(4.67) | 1791(4.72) | 30(2.22) |  |
| Other Race | 2087(5.74) | 2049(5.73) | 38(6.05) |  |
| **Marital Status** |  |  |  | 0.38 |
| non-single | 15797(66.21) | 15404(66.24) | 393(64.26) |  |
| single | 9461(33.79) | 9181(33.76) | 280(35.74) |  |
| **Education** |  |  |  | < 0.0001 |
| <high school | 1963(3.54) | 1876(3.47) | 87(7.15) |  |
| high school | 8862(31.89) | 8572(31.64) | 290(44.32) |  |
| >high school | 14433(64.57) | 14137(64.88) | 296(48.52) |  |
| **Diabetes** |  |  |  | < 0.0001 |
| No | 21643(89.53) | 21194(89.88) | 449(71.84) |  |
| Yes | 3615(10.47) | 3391(10.12) | 224(28.16) |  |
| **Hypertension** |  |  |  | < 0.0001 |
| No | 15368(65.82) | 15226(66.61) | 142(25.48) |  |
| Yes | 9890(34.18) | 9359(33.39) | 531(74.52) |  |

Abbreviations: OBS, oxidation balance score; PIR, family income to poverty.

**Table S2**. Number of individuals in each group and range of values according to quartiles for OBS.

|  | **Q1** | **Q2** | **Q3** | **Q4** |
| --- | --- | --- | --- | --- |
| **OBS** | | | | |
| N | 6190 | 6406 | 5808 | 6854 |
| Value | 3.00-15.00 | 16.00-21.00 | 22.00-26.00 | 27.00-38.00 |
| **OBS.dietary** | | | | |
| N | 5717 | 6516 | 6140 | 6885 |
| Value | 1.00-10.00 | 11.00-16.00 | 17.00-21.00 | 22.00-31.00 |
| **OBS.lifestyle** | | | | |
| N | 5861 | 4986 | 5079 | 9332 |
| Value | 0.00-3.00 | 4.00-4.00 | 5.00-5.00 | 6.00-8.00 |

Abbreviations: OBS, oxidation balance score.

**Table S3**. Association between OBS in tertiles and prevalence of stroke.

|  | **Crude model**  **OR (95%CI) P-value** | **Adjusted model 1**  **OR (95%CI) P-value** | **Adjusted model 2**  **OR (95%CI) P-value** |
| --- | --- | --- | --- |
| **OBS.DIETARY** | 0.97 (0.96, 0.98) <0.0001 | 0.98 (0.96, 1.00) 0.0164 | 0.98 (0.96, 0.99) 0.0128 |
| **OBS.DIETARY tertile** |  |  |  |
| Low | Ref. | Ref. | Ref. |
| Middle | 0.88 (0.69, 1.13) 0.3283 | 1.00 (0.75, 1.33) 0.9881 | 0.99 (0.74, 1.32) 0.9401 |
| High | 0.66 (0.52, 0.82) 0.0003 | 0.82 (0.61, 1.11) 0.2051 | 0.81 (0.60, 1.10) 0.1771 |
| p for trend | 0.0002 | 0.1978 | 0.1707 |
| **OBS.LIFESTYLE** | 1.01 (0.95, 1.07) 0.7358 | 0.92 (0.86, 0.98) 0.0119 | 0.96 (0.89, 1.02) 0.2011 |
| **OBS.LIFESTYLE tertile** |  |  |  |
| Low | Ref. | Ref. | Ref. |
| Middle | 1.28 (0.97, 1.69) 0.0841 | 0.93 (0.69, 1.25) 0.6146 | 0.99 (0.73, 1.33) 0.9367 |
| High | 1.08 (0.80, 1.47) 0.6039 | 0.70 (0.51, 0.96) 0.0284 | 0.81 (0.59, 1.12) 0.2078 |
| p for trend | 0.8059 | 0.0122 | 0.1374 |
| **OBS** | 0.97 (0.96, 0.98) <0.0001 | 0.97 (0.96, 0.99) 0.0017 | 0.97 (0.96, 0.99) 0.0019 |
| **OBS tertile** |  |  |  |
| Low | Ref. | Ref. | Ref. |
| Middle | 0.81 (0.62, 1.06) 0.1261 | 0.86 (0.64, 1.15) 0.3027 | 0.85 (0.64, 1.14) 0.2935 |
| High | 0.67 (0.54, 0.82) 0.0002 | 0.76 (0.58, 0.99) 0.0419 | 0.76 (0.58, 0.99) 0.0429 |
| p for trend | 0.0002 | 0.0425 | 0.0437 |

Abbreviations: OBS, oxidation balance score. The crude model was not adjusted for any covariates. Adjusted model 1 was adjusted for age, gender, race, marital status, PIR, education level, and total energy intake. Adjusted model 2 was further adjusted for diabetes and hypertension based on adjusted model 1.

**Table S4**. Association between OBS in quintiles and prevalence of stroke.

|  | **Crude model**  **OR (95%CI) P-value** | **Adjusted model 1**  **OR (95%CI) P-value** | **Adjusted model 2**  **OR (95%CI) P-value** |
| --- | --- | --- | --- |
| **OBS.DIETARY** | 0.97 (0.96, 0.98) <0.0001 | 0.98 (0.96, 1.00) 0.0164 | 0.98 (0.96, 0.99) 0.0128 |
| **OBS.DIETARY quintiles** |  |  |  |
| Q0 | Ref. | Ref. | Ref. |
| Q1 | 0.79 (0.59, 1.05) 0.1066 | 0.84 (0.62, 1.14) 0.2781 | 0.83 (0.61, 1.13) 0.2388 |
| Q2 | 0.70 (0.51, 0.97) 0.0355 | 0.79 (0.55, 1.13) 0.1973 | 0.78 (0.54, 1.12) 0.1766 |
| Q3 | 0.61 (0.46, 0.82) 0.0012 | 0.73 (0.52, 1.03) 0.0745 | 0.72 (0.51, 1.01) 0.0557 |
| Q4 | 0.56 (0.42, 0.75) 0.0002 | 0.70 (0.48, 1.02) 0.0652 | 0.68 (0.46, 1.00) 0.0518 |
| p for trend | <0.0001 | 0.0540 | 0.0427 |
| **OBS.LIFESTYLE** | 1.01 (0.95, 1.07) 0.7358 | 0.92 (0.86, 0.98) 0.0119 | 0.96 (0.89, 1.02) 0.2011 |
| **OBS.LIFESTYLE quintiles** |  |  |  |
| Q0 | Ref. | Ref. | Ref. |
| Q1 | 1.21 (0.76, 1.91) 0.4171 | 0.96 (0.60, 1.52) 0.8534 | 0.98 (0.62, 1.57) 0.9490 |
| Q2 | 1.43 (0.95, 2.15) 0.0902 | 0.99 (0.64, 1.51) 0.9510 | 1.04 (0.68, 1.60) 0.8576 |
| Q3 | 1.40 (0.91, 2.16) 0.1306 | 0.83 (0.52, 1.32) 0.4284 | 0.92 (0.57, 1.47) 0.7312 |
| Q4 | 1.20 (0.79, 1.81) 0.3913 | 0.68 (0.44, 1.05) 0.0817 | 0.80 (0.52, 1.24) 0.3265 |
| p for trend | 0.6142 | 0.0097 | 0.1275 |
| **OBS** | 0.97 (0.96, 0.98) <0.0001 | 0.97 (0.96, 0.99) 0.0017 | 0.97 (0.96, 0.99) 0.0019 |
| **OBS quintiles** |  |  |  |
| Q0 | Ref. | Ref. | Ref. |
| Q1 | 0.72 (0.53, 0.96) 0.0261 | 0.68 (0.50, 0.93) 0.0161 | 0.68 (0.50, 0.92) 0.0147 |
| Q2 | 0.77 (0.57, 1.05) 0.1042 | 0.78 (0.55, 1.10) 0.1639 | 0.78 (0.55, 1.10) 0.1517 |
| Q3 | 0.73 (0.54, 0.99) 0.0417 | 0.76 (0.54, 1.07) 0.1170 | 0.75 (0.53, 1.06) 0.1086 |
| Q4 | 0.50 (0.38, 0.68) <0.0001 | 0.53 (0.36, 0.77) 0.0012 | 0.53 (0.36, 0.77) 0.0012 |
| p for trend | <0.0001 | 0.0077 | 0.0081 |

Abbreviations: OBS, oxidation balance score. The crude model was not adjusted for any covariates. Adjusted model 1 was adjusted for age, gender, race, marital status, PIR, education level, and total energy intake. Adjusted model 2 was further adjusted for diabetes and hypertension based on adjusted model 1.
